# Long-term liming changes pasture mineral profile

**DOI:** 10.1038/s41598-024-53908-1

**Published:** 2024-02-12

**Authors:** Guangdi D. Li, Mark K. Conyers, Gordon Refshauge, Forough Ataollahi, Richard C. Hayes

**Affiliations:** 1 NSW Department of Primary Industries, Wagga Wagga Agricultural Institute, Wagga Wagga, NSW 2650 Australia; 2 NSW Department of Primary Industries, Cowra Agricultural Research and Advisory Station, Cowra, NSW 2794 Australia

**Keywords:** Element cycles, Systems analysis, Plant development

## Abstract

There is limited information on changes of pasture mineral concentrations over the long-term in response to liming. A long-term field experiment was conducted to assess the influence of lime application on (a) changes in pasture mineral composition over time; and (b) key pasture mineral concentrations and ratios important to animal health. Perennial and annual pastures with or without lime application were sampled annually over 12 years and analysed for macro- and micro-minerals. Mineral ratios and indices were calculated to assess the potential impact on animal health. Liming increased the concentrations of calcium, sodium and silicon, but decreased the concentrations of micro-nutrients including copper, zinc and manganese. The same trend was found in both annual and perennial pastures although there were some fluctuations between years. Liming increased the calcium:phosphorus ratio and the dietary cation–anion difference but reduced the tetany index on both annual and perennial pastures. These findings suggest a potential benefit to improve animal health outcomes for some disorders on the limed pastures. However, the reduced concentrations of some trace elements following liming potentially decreases antioxidant capacity and requires further research.

## Introduction

Liming is the most effective management practice to slow or reverse soil acidification^1^. Liming acidic soils is known to change soil chemical, physical and biological properties and generally improve soil health and function^1–3^. The cost of liming can be recovered with cropping in a relatively short timeframe^4^ although returns vary with crop type^5^. However, it is more difficult to demonstrate economically viable responses to lime in pasture and animal production^6,7^.

Positive yield responses to lime are often observed in crops^8^ and pastures^9^, with differences in pasture botanical composition improving feed quality by favouring desirable pasture species^10,11^. Ultimately, limed pastures have been shown to produce more meat and/or wool^12,13^, but there has been little attention paid to the direct effect of lime on the mineral profile of pastures.

The essential minerals required to maintain function in productive livestock is ultimately supplied through their diet. The abundance of a mineral available to livestock is governed by a range of factors including the quantum of that mineral in the forage and mineral interactions that may impact mineral absorption by the animal. Table 1 summarised requirements of the major and minor minerals for liveweight gain, pregnancy and lactation in sheep, cattle and goats, adopted from NRC^14,15^. There are reports of hypomagnesemia in grazing livestock caused by either low magnesium (Mg) concentration in forage or by a mineral imbalance such as high potassium (K) and low sodium (Na) in which excess K inhibits absorption of Mg in the rumen^16,17^. The calculated ratios or indices of these minerals are considered as important indicators of metabolic disorders in grazing livestock which identify the need for mineral supplementation^18,19^. Moreover, mineral requirements (Table 1) provide valuable information about known toxicities or deficiencies associated with particular macro- and micro-nutrients.

**Table 1:** Required level of major minerals including calcium (Ca), potassium (K), magnesium (Mg), sodium (Na), phosphorus (P) and sulphur (S) (g/kg of daily DM intake), and minor minerals including copper (Cu), iron (Fe), manganese (Mn) and zinc (Zn) (mg/kg of daily intake) for growing, pregnancy and lactation in sheep, beef cattle and meat goats.

Livestock		Ca	K	Mg	Na	P	S	Cu	Fe	Mn	Zn
Sheep	Growing^a^	3.1	4.4	0.9	0.6	2.5	1.8	7	78	26	45
	Late pregnancy^b^	4.9	4.5	1.0	0.5	2.9	1.6	11	51	38	51
	Early lactation^c^	4.1	5.5	1.3	0.7	3.5	1.6	11	13	25	79
Cattle^d^	Growing	3.6	6.0	1.0	0.7	1.9	1.5	10	50	20	30
	Gestation	3.7	6.0	1.2	0.7	1.8	1.5	10	50	40	30
	Early lactation	5.3	7.0	2.0	1.0	3.3	1.5	15	50	40	30
Goats	Growing^a^	6.0	4.9	0.8	0.9	2.9	2.7	17	34	12	11
	Late pregnancy^b^	4.4	6.1	0.8	0.7	2.2	2.9	27	75	44	59
	Early lactation^c^	5.8	9.0	0.9	0.8	3.4	2.6	35	26	26	82

^a^40 kg lamb at 4 months of age, gaining 250 g/day (Tables 15-2 and 15-3^14^) or a 20 kg meat goat (kid) gaining 100 g/day (Tables 15-5 and 15-7^14^), adjusted for dry matter intake.

^b^60 kg twin-bearing mature ewe at late pregnancy (Tables 15-1 and 15-3^14^), or 50 kg twin-bearing non-dairy meat doe (Tables 15-4 and 15-7^14^), adjusted for dry matter intake.

^c^60 kg twin-bearing mature ewe (Tables 15-1 and 15-3^14^), or twin-bearing mature meat doe (Tables 15-4 and 15-7^14^), adjusted for dry matter intake.

^d^Beef cattle nutrient concentration requirements taken from Table 5-1^15^.

The addition of lime to an acidic soil is known to change the pasture mineral profile. There are a number of mechanisms driving these changes, including (i) the application of large quantities of calcium (Ca) in lime; (ii) a change in soil pH affecting the availability of multiple minerals^1^; (iii) increases in plant growth in some species which may enable greater root exploration of the soil volume leading to increased access to a minerals (especially micro-nutrients) in the soil^20^, and (iv) dilution of some nutrient contents in association with the increased dry matter or maturity^21^. Hayes et al*.*^22^ evaluated the change in pasture mineral profile following liming in a range of species in south-eastern Australia and demonstrated that the addition of lime impacted the concentration of over a dozen minerals. However, in a later experiment at the same site, Hayes et al*.*^23^ found no effect of lime on the mineral profile of pasture herbage other than a reduction in manganese (Mn) concentrations. The disparity in results from these two studies at the same site highlights an inconsistent response of pasture mineral composition to liming on acidic soils.

In the present study, pasture mineral concentrations were monitored over 12 years in a long-term liming experiment. We hypothesized that (a) liming changes pasture mineral composition over the long term; and (b) liming alters key pasture mineral ratios, and potentially reducing the risk of metabolic disorders in livestock.

## Results

### Rainfall

**Figure 1:**
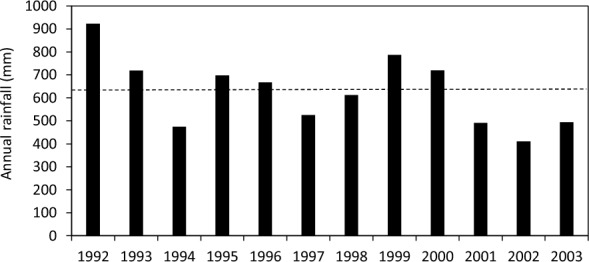
Annual rainfall (bars) and the average rainfall (dotted line) in 1992–2003 at the experimental site.

The average rainfall was 627 mm from 1992 to 2003 at the experimental site. The site had experienced 4 drought years with rainfall below decile 2 in 1994 (475 mm), 2001 (492 mm), 2002 (410 mm) and 2003 (494 mm), and 4 wet years with rainfall above decile 7 in 1992 (923 mm), 1993 (719 mm), 1999 (787 mm) and 2000 (720 mm) (Fig. 1).

### Macro elements in plants

**Figure 2:**
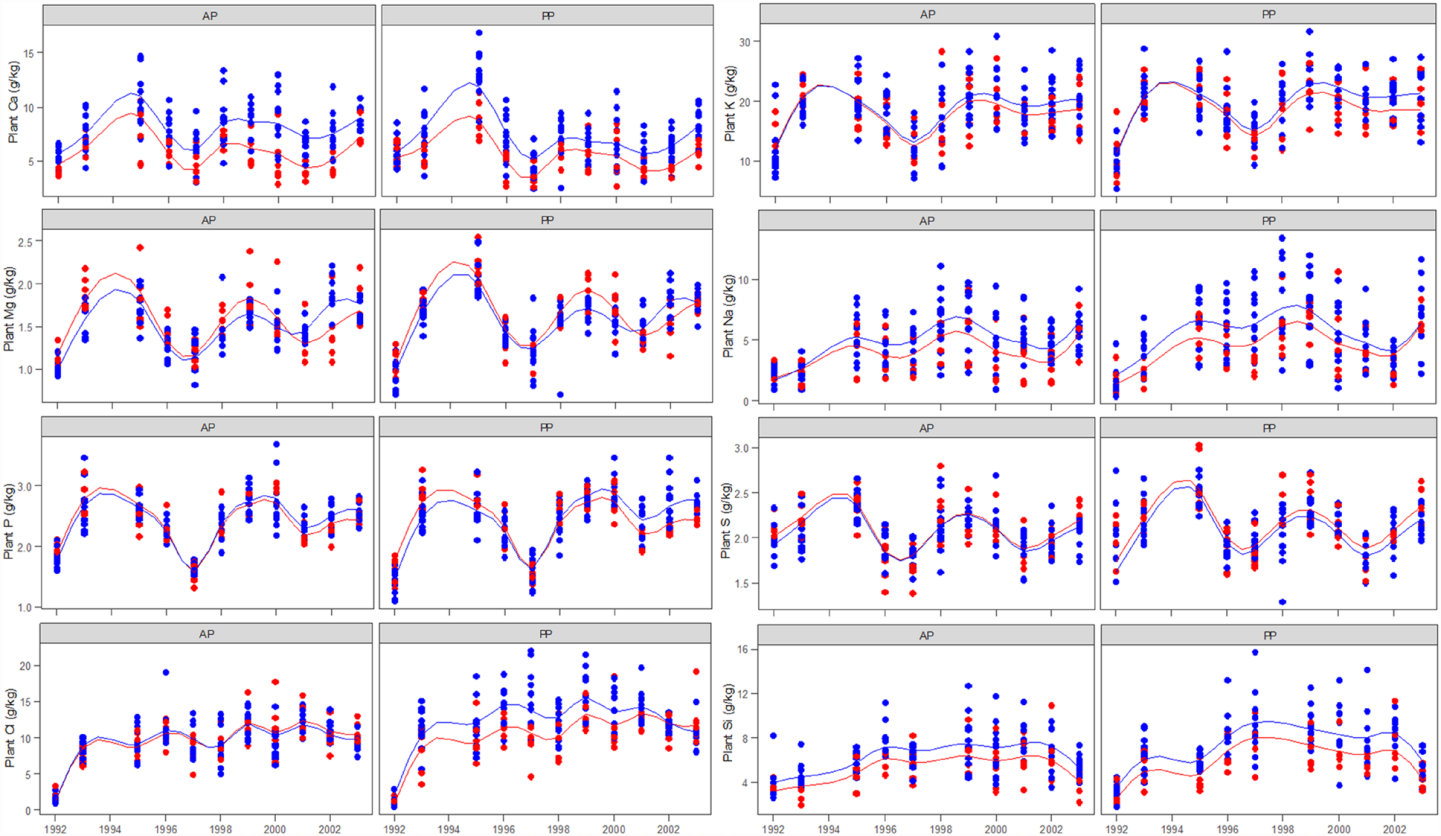
Pasture mineral concentration at anthesis for macro elements (Ca, calcium; K, potassium; Mg, magnesium; Na, sodium; P, phosphorus; S, sulphur; and Cl, chloride) over 12 years on limed () and unlimed () treatments in annual (AP) and perennial pastures (PP). The lines are spline-fitted with corresponding treatments. No plant samples were taken in 1994 due to the extreme drought condition.

*Calcium*. There were significant differences in the Ca concentration between lime treatments (*P* < 0.01) and pasture types (*P* < 0.05) with no interaction between lime and pasture type (Table 2). However, there was a significant pasture and year interaction (*P* < 0.01). Lime increased the Ca concentration in both annual and perennial pastures 6 months after lime was applied and Ca concentration in pasture was maintained at a higher level throughout the experimental period (Fig. 2). Perennial pastures had a lower Ca concentration than annual pastures in the second liming cycle from year 7 onwards, particularly on the limed treatment (Fig. 2). In most years, the Ca concentration was above the nutrient requirement for growing, pregnant or lactating sheep, cattle and goats (Table 1) under both limed and unlimed treatments for both pasture types. Exceptions included late pregnancy sheep, early lactation cattle and goats and growing goats, particularly under the unlimed treatments. Perennial pasture failed to supply the mineral concentration requirement for growing and early lactation goats and early lactation cattle in more years than the annual pasture (Fig. 2).

**Table 2:** Wald statistics for fixed effects and their interactions for pasture mineral concentrations for macro elements, micro elements, mineral ratios and indices over 12 years.

Strata/decomposition	Effect^a^	Ca	K	Mg	Na	P	S	Cl	Si	Cu	Fe	Mn	Zn	Al	K:Na^d^	K:(Na + Mg )^e^	Ca:P^f^	Tetany^g^	DCAD^h^
		Macro element^b^								Micro element^c^									
Experimental unit																			
Lime	F	54.1**	1.9n.s	2.2n.s	5.7*	0.4n.s	2.4n.s	2.9n.s	43.5***	11.5***	0.2n.s	70.4***	55.9***	0.4n.s	1.1n.s	0.5n.s	28.1***	9.0**	43.8***
Pasture	F	5.7*	7.6**	12.0***	9.9**	0.6n.s	8.4**	13.6***	27.5***	0.0n.s	2.1n.s	1.4n.s	0.1n.s	1.0n.s	0.3n.s	0.5n.s	6.4*	11.5***	0.1n.s
Lime × Pasture	F	0.3n.s	0.4n.s	0.1n.s	0.1n.s	0.1n.s	0.2n.s	3.6 (*P* = 0.06)	0.7n.s	5.8*	0.2n.s	0.5n.s	1.0n.s	1.8n.s	0.1n.s	0.3n.s	0.2n.s	1.2n.s	3.1n.s
Residual	R																		
Experimental unit × Year																			
Year	F	0.9n.s	56.8***	91.3***	60.4***	166.3***	3.4n.s	287.8***	78.9***	114.2***	7.6***	92.9***	114.5***	37.0***	25.9***	7.7***	36.3***	22.1***	18.9***
Lime × Year	F	1.8n.s	4.0*	17.7***	0.1n.s	33.6***	0.1n.s	4.4*	0.8n.s	0.0n.s	5.8*	16.7***	0.3n.s	1.0n.s	0.4n.s	0.0n.s	1.1n.s	0.4n.s	23.5***
Pasture × Year	F	10.2**	0.9n.s	0.0n.s	0.6n.s	16.1***	0.8n.s	0.8n.s	0.3n.s	5.1*	0.6n.s	2.3n.s	2.8n.s	0.2n.s	0.1n.s	0.7n.s	21.5***	3.6n.s	1.4n.s
Lime × Pasture × Year	F	0.9n.s	0.1n.s	1.6n.s	2.0n.s	4.6*	0.5n.s	0.4n.s	0.0n.s	0.2n.s	0.0n.s	0.7n.s	0.1n.s	1.2n.s	2.1n.s	1.8n.s	2.3n.s	0.5n.s	1.0n.s
Residual	R																		

^a^F, Fixed effect; R, Random effect; *P < 0.05; **P < 0.01; ***P < 0.001; ns, not significant.

^b^Macro elements: Ca, calcium; K, potassium; Mg, magnesium; Na, sodium; P, phosphorus; S, sulphur; and Cl, chloride.

^c^Micro elements: Al, aluminium; Mn, manganese; Cu, cupper; Fe, iron; Zn, zinc; and Si, silicon.

^d^K:Na = K/Na ratio^29^.

^e^K/(Na + Mg) (milliequivalent) = (K/0.039)/[(Na/0.023) + (Mg/0.012)]^29^.

^f^Ca:P = Ca/P ratio^29^.

^g^Tetany Index (milliequivalent) = (K/0.039)/[(Ca/0.02) + (Mg/0.012)]^29^.

^h^DCAD (dietary cation–anion difference, milliequivalent/100 g) = DCAD = [(Na/0.023 + K/0.039) − (Cl/0.0355 + S/0.016)]^29^.

*Potassium*. There was no significant difference in the K concentration between lime treatments, although both limed annual and perennial pastures tended towards a slightly higher K concentration in the second liming cycle due to significant lime and year interaction (*P* < 0.05, Table 2). The K concentration was higher in perennial pastures compared to annual pastures (*P* < 0.01, Table 2). The K concentration was above the nutrient concentration requirement for growing, pregnant or lactating sheep, cattle and goats (Table 1) under both limed and unlimed treatments for both pasture types (Fig. 2).

*Magnesium.* Perennial pastures had a higher Mg concentration compared with annual pastures (*P* < 0.001, Table 2 and Fig. 2). There was no difference in the Mg concentration between limed and unlimed treatments (Table 2). However, due to a strong interaction between lime and year (*P* < 0.001), Mg concentration was lower on the limed treatments in most years but higher in the last 3 years, compared to that on the unlimed treatments for both pasture types (Fig. 2). The Mg concentration was above the nutrient concentration requirement for growing, pregnant or lactating sheep, cattle and goats (Table 1) under both limed and unlimed treatments for both pasture types. Sheep requirements in early lactation were not met in 1997 in the annual pastures, but were met by the perennial pastures, whereas cattle requirements in early lactation were only met in 1994 and 1995 in perennial pastures (Fig. 2).

*Sodium.* There were significant differences in the Na concentration between lime treatments (*P* < 0.05), and between pasture types (*P* < 0.01, Table 2). There was also a strong year effect on Na concentration of pastures (*P* < 0.001). The limed treatment had a higher Na concentration than the unlimed treatment (Fig. 2), and perennial pastures had a higher Na concentration than annual pastures (Fig. 2). The Na concentration was above the nutrient concentration requirement for growing, pregnant or lactating sheep, cattle and goats (Table 1) under both limed and unlimed treatments for both pasture types (Fig. 2).

*Phosphorus (P).* There was no significant main effect of lime or pasture type on the P concentration in herbage, however, both lime × year and pasture × year interactions were highly significant (*P* < 0.001) due to the strong year effect (Table 2). In general, the P concentration on the limed treatments was lower during the first liming cycle, but higher in the second liming cycle, particularly at the last 3 years compared to those on the unlimed treatments (Fig. 2). The P concentration was below the nutrient concentration requirement for growing sheep, late pregnancy in sheep, early lactation in sheep, early lactation cattle and growing, pregnancy and lactation goats (Table 1) under both limed and unlimed treatments for both pasture types. Liming helped meet P requirements in the perennial pasture for growing goats for most of the years (Fig. 2).

*Sulphur (S).* There was a significant effect of pasture type on the S concentration, but no lime effect, year effect or associated interactions (Table 2). The S concentration was slightly higher, in general, on the perennial pastures than on the annual pastures, but was marginally below requirements for growing, pregnant or lactating goats (Table 1) for most years regardless of liming, but did meet the requirements for sheep and cattle (Fig. 2).

*Chloride (Cl).* There was a weak interaction (*P* = 0.06) between lime and pasture type (Table 2) with a strong pasture effect (*P* < 0.001). Perennial pastures on the limed treatments had higher Cl concentrations in most of years than the unlimed treatments, but no difference was found between limed and unlimed treatments on annual pastures (Fig. 2).

*Silicon (Si).* There were differences in the Si concentration between lime treatments (*P* < 0.001), and between pasture types (*P* < 0.001) (Table 2). There was also a strong year effect on the Si concentration in pastures (*P* < 0.001). The limed treatments had a higher Si concentration than unlimed treatments and the Si concentration was higher in perennial compared to annual pastures (Fig. 2). Authors were not able to find relevant information on nutrient requirement of sheep, cattle and goat for Si.

### Micro elements in plants

**Figure 3:**
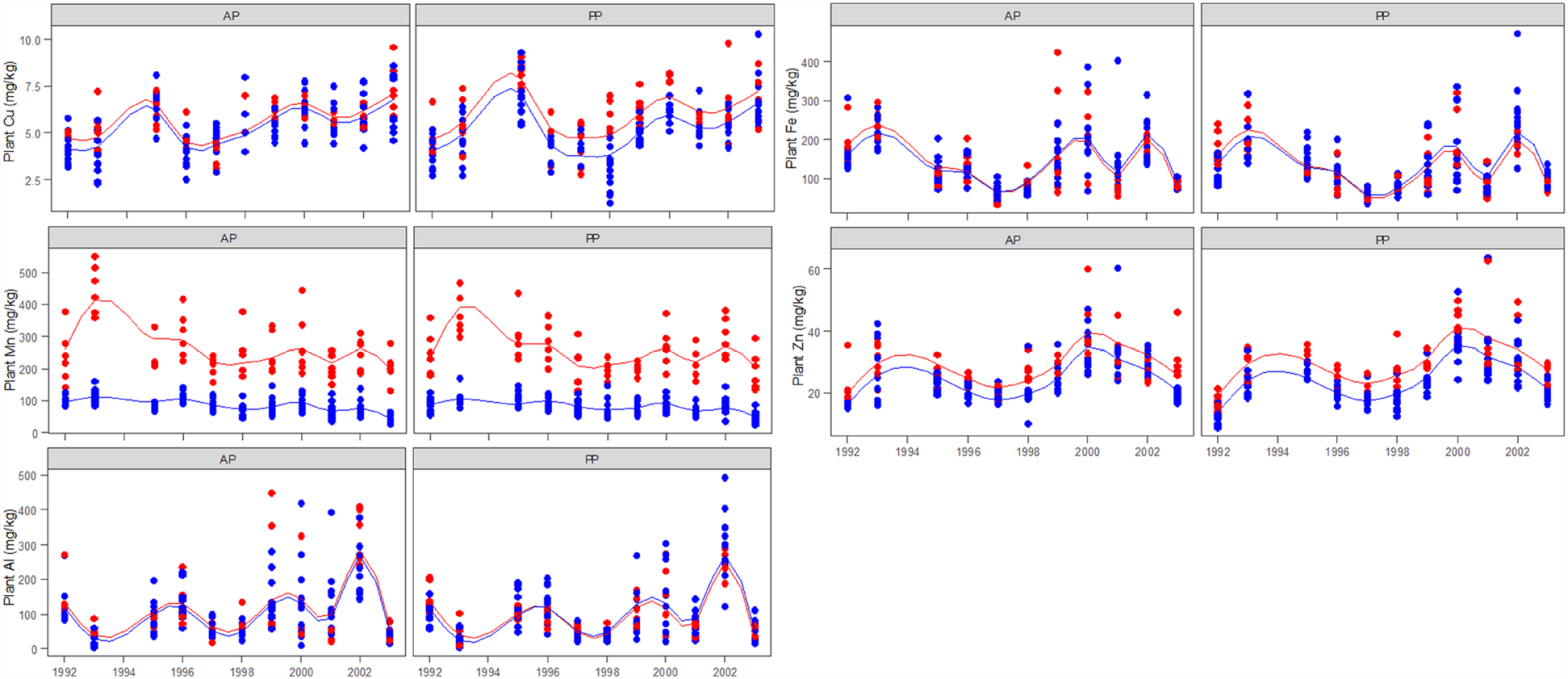
Pasture mineral concentration at anthesis for micro elements (Al, aluminium; Mn, manganese; Cu, copper; Fe, iron; and Zn, zinc) over 12 years on limed () and unlimed () treatments in annual (AP) and perennial pastures (PP). The lines are spline-fitted with corresponding treatments. No plant samples were taken in 1994 due to the extreme drought condition.

*Copper (Cu).* In general, the Cu concentration was greater on the unlimed than on the limed treatments, particularly on perennial pastures (*P* < 0.001, Table 2). There was a pasture and year interaction with higher Cu concentrations on perennial pastures than on annual pastures during the first liming cycle but no difference between pasture types during the last 4 years of the experiment (Fig. 3). In most years, the Cu concentration was below the nutrient concentration requirement for cattle or goats for all classes, and did not meet sheep Cu requirements in late pregnancy or early lactation (Table 1) under both limed and unlimed treatments and both pasture types (Fig. 3).

*Iron (Fe).* There was no lime nor pasture effect on the Fe concentration (*P* > 0.05) but there was a strong year effect (*P* < 0.001) (Table 2). There was a lime and year interaction (*P* < 0.05) with slightly lower Fe concentrations in the first liming cycle, but slightly higher Fe concentration in the second liming cycle on the limed compared with the unlimed treatments (Fig. 3). There was no difference in Fe concentration between annual and perennial pastures (Table 2). The Fe concentration was generally above the nutrient concentration requirement for growing, pregnant or lactating sheep, cattle and goats (Table 1) under both limed and unlimed treatments and both pasture types. In 1997 and 1998 the requirements for growing lambs or late pregnancy goats were not met, or were marginal (Fig. 3).

*Manganese (Mn).* There was a strong lime effect on the Mn concentration (*P* < 0.001) with significantly lower Mn concentrations on the limed treatments on both pasture types (Table 2). There was no difference in Mn concentration between annual and perennial pastures. The significant lime and year interaction was due to strong inter-annual variation with the greatest Mn concentration observed in 1993 (Fig. 3). The Mn concentration was above the nutrient concentration requirement for growing, pregnant or lactating sheep and cattle (Table 1) under both limed and unlimed treatments and both pasture types although the final year of sampling the mineral concentration in limed pastures was marginal for late pregnant goats (Fig. 3).

*Zinc (Zn).* There was a strong lime effect with lower Zn concentrations on the limed compared with unlimed treatments (*P* < 0.001, Table 2). There was a strong year effect with two peaks in 1993 and 2000, but no difference was found between annual and perennial pastures (Fig. 3). The Zn concentration was below the nutrient concentration requirement for sheep and particularly for cattle. It did not meet the requirements for late pregnancy or early lactation goats (Table 1) under both limed and unlimed treatments and both pasture types. Lime increased the number of years when cattle requirements were not met under both pasture types (Fig. 3).

*Aluminium (Al).* There was a strong year effect on the Al concentration (*P* < 0.001) with no lime nor pasture effects (Table 2). There were 4 peaks and 4 troughs for the Al concentration over the 12 years with the highest Al concentration observed in 2002 (Fig. 3). Mineral concentration requirements are not described, but the maximum tolerable concentration for sheep, cattle and goats (1000 mg/kg^18^) was not exceeded at any time (Fig. 3).

### Mineral ratios and indices

**Figure 4:**
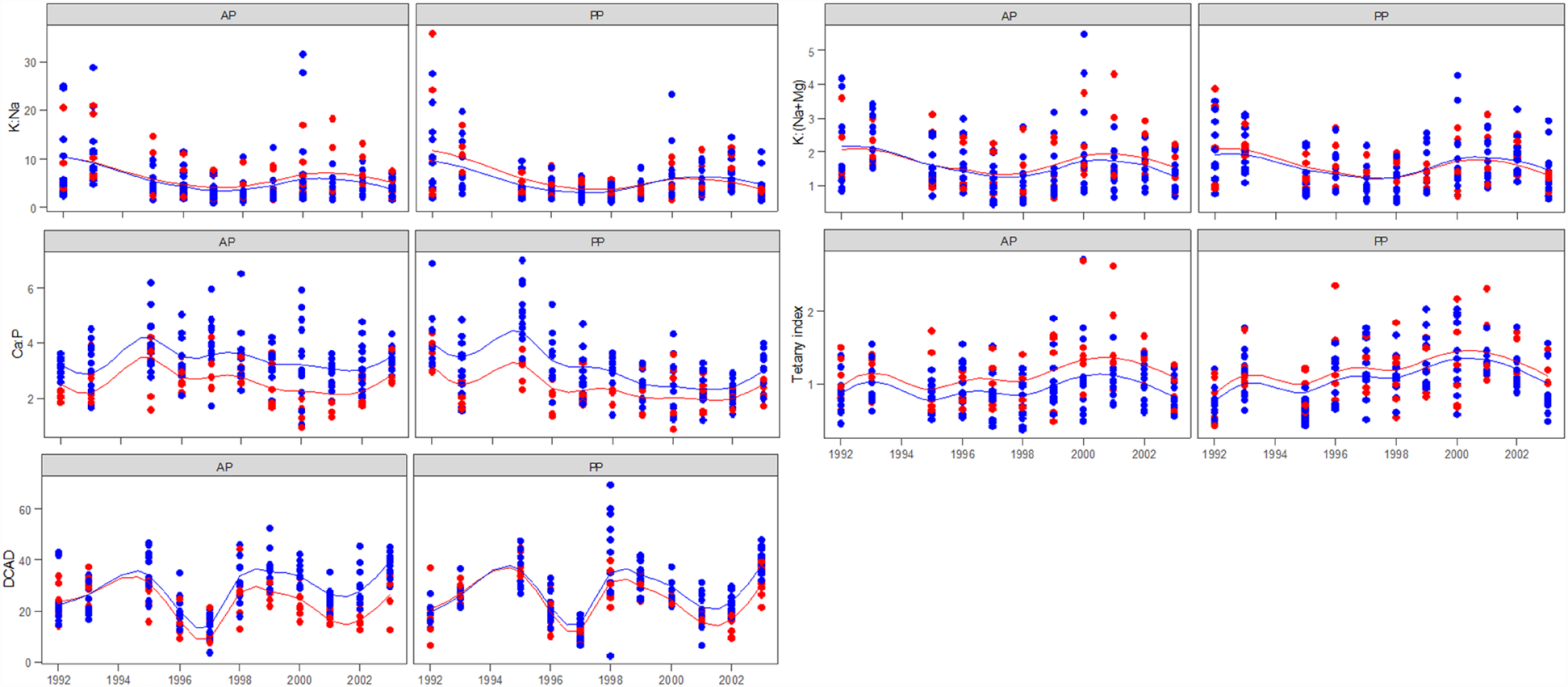
Pasture mineral concentration ratios and indices [K:Na, K:(Na + Mg), Ca:P ratio, grass tetany index and dietary cation–anion difference (DCAD)] with over 12 years on limed () and unlimed () treatments in annual (AP) and perennial pastures (PP). The lines are spline-fitted with corresponding treatments. No plant samples were taken in 1994 due to the extreme drought condition.

*K:Na and K:(Na *+ *Mg) ratios.* There was no significant lime nor pasture effects on the K:Na ratio, but there was a strong year effect (Table 2). The K:Na ratio was greater in the first two years, decreased to the lowest value in 1997, then increased slightly to reach a plateau from 2000 (Fig. 4). The K:(Na + Mg) ratio followed the same trend as the K:Na ratio (Table 2 and Fig. 4). The K:(Na + Mg) ratio was under maximum tolerant limit (< 6 mEq) suggested by Dove and Kelman^24^.

*Ca:P ratio*. There were significant differences in the Ca:P ratio between lime treatments (*P* < 0.001) and pasture types (*P* < 0.05) with no interaction between lime and pastures (Table 2). Lime increased the Ca:P ratio significantly on both annual and perennial pastures over the whole experimental period. However, there was a significant pasture × year interaction (*P* < 0.001) with higher Ca:P ratio in the first two years, but lower from 1996 onwards on the limed perennial pastures (Fig. 4). The Ca:P ratio was above the recommended ratio (1.1–2.1% DM) for these ruminants^19,25,26^ under both limed and unlimed treatments and both pasture types (Fig. 4).

*Tetany index.* The tetany index was lower on the limed treatment compared to unlimed treatments and with slightly higher value on perennial pastures compared to annual pastures throughout the experimental period (*P* < 0.05, Table 2 and Fig. 4). There was also a strong effect of time with the tetany index observed to be higher in the second liming cycle compared to the first liming cycle (*P* < 0.001, Table 2). The tetany index was below the recommended limit (< 2.2 mEq) for ruminants^19,25^ under both limed and unlimed treatments and both pasture types.

*Dietary cation–anion difference.* There was a strong lime and time interaction on DCAD (*P* < 0.001, Table 2 and Fig. 4), with no effect of pasture type observed (*P* > 0.05). There was large inter-annual variation with two troughs observed in 1997 and 2001 (Fig. 4), associated with dry seasons (Fig. 1). There was little difference in DCAD between lime treatments initially but as the experiment progressed, DCAD values increased in the limed compared to unlimed treatments. DCAD was above the recommended limit (< 12 meq per 100 g DM) for ruminants^19,25^ under both limed and unlimed treatments and both pasture types.

### Correlations between plant elements in herbage

**Figure 5:**
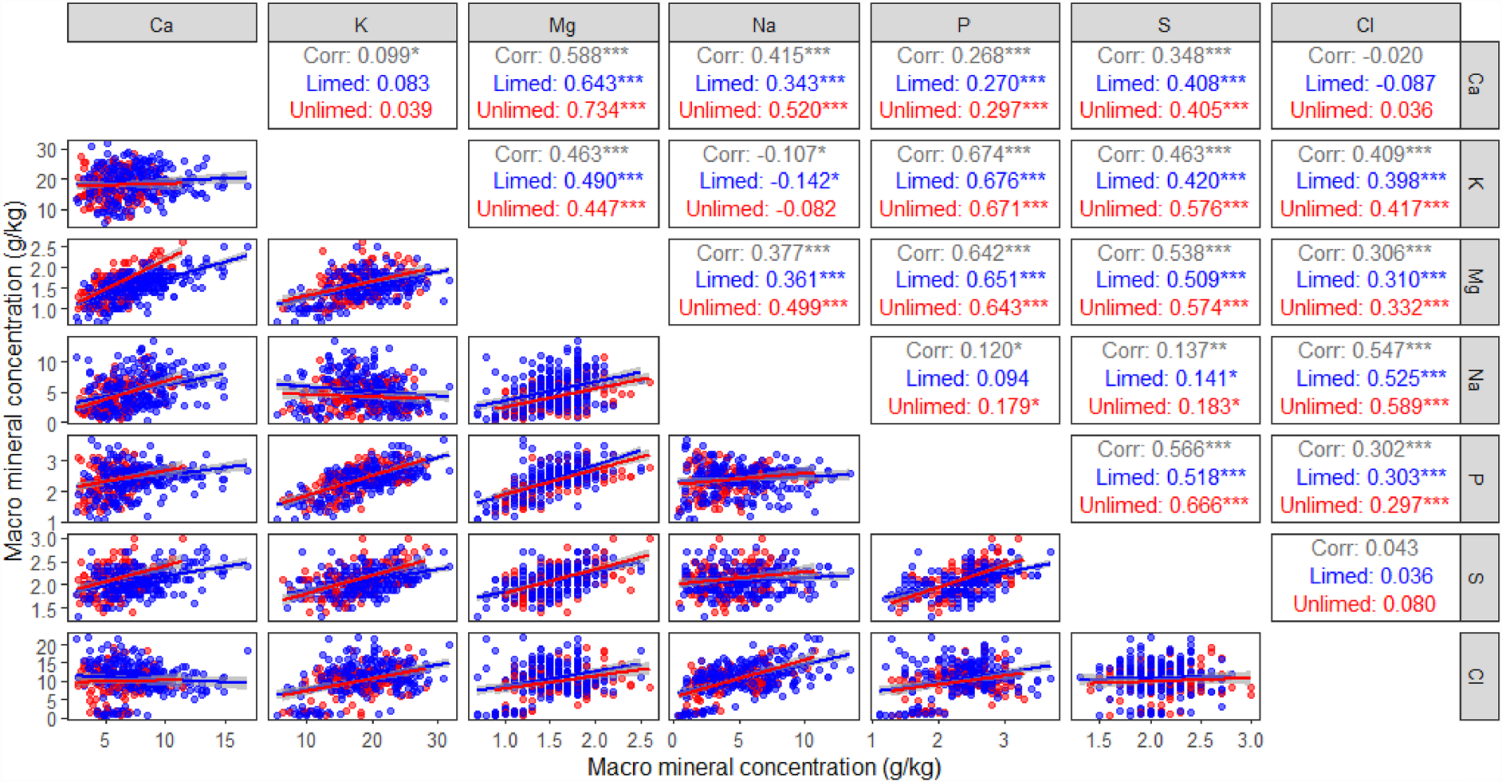
Correlations between pasture macro elements (Ca, calcium; K, potassium; Mg, magnesium; Na, sodium; P, phosphorus; S, sulphur; and Cl, chloride) at anthesis. Coloured lines are fitted regression lines between each pair of plant elements under limed () and unlimed () treatments. *P < 0.05; **P < 0.01; ***P < 0.001.

For macro elements, Ca was positively correlated with Mg, Na, P and S on both limed and unlimed treatments, but there was no correlation between Ca and either K or Cl (Fig. 5). Potassium was positively correlated with Mg, P, S and Cl on both limed and unlimed treatments, but negatively correlated with Na only on the limed treatment. Magnesium was positively correlated with Na, P, S and Cl on both limed and unlimed treatments. Sodium was positively correlated with S and Cl on both limed and unlimed treatments, and with P only on the unlimed treatments (Fig. 5). Phosphorus was positively correlated with S and Cl on both limed and unlimed treatments. There was no correlation between S and Cl on either limed or unlimed treatments (Fig. 5).

**Figure 6:**
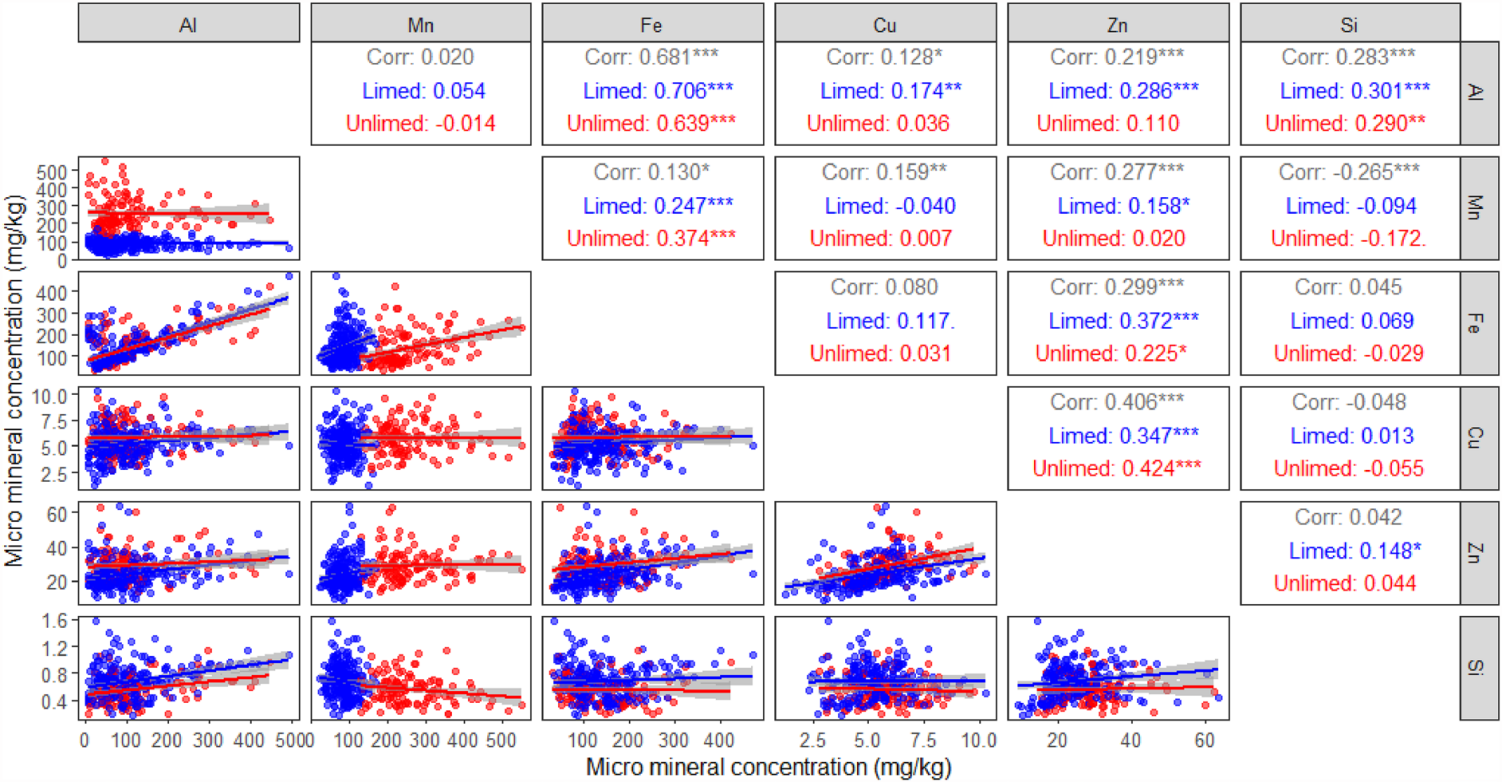
Correlations between pasture micro elements (Al, aluminium; Mn, manganese; Cu, copper; Fe, iron; Zn, zinc; and Si, silicon) at anthesis. Coloured lines are fitted regression lines between each pair of plant elements under limed () and unlimed () treatments. *P < 0.05; **P < 0.01; ***P < 0.001.

For micro elements, Al was positively correlated with Fe and Si on both limed and unlimed treatments, and positively correlated with Cu and Zn only on the limed treatment, but not on the unlimed treatment (Fig. 6). Mn was not correlated with Al, Cu and Si on either limed or unlimed treatments, but positively correlated with Fe on both limed and unlimed treatments and weakly correlated to Zn on the limed treatment. Fe was positively correlated with Zn on both limed and unlimed treatments, but not with Cu and Si for either treatment. Copper was highly correlated to Zn on both limed and unlimed treatments, but not with Si, while Zn was weakly correlated to Si on the limed treatment only (Fig. 6).

Liming had no effect on the concentration of either Al or Fe in either pasture type (Fig. 3) despite liming changed the solubility of these elements in soil. Further, Al and Fe were highly correlated at higher concentrations rather than at low concentrations (Fig. 6).

## Discussion

The results support the hypothesis that ongoing application of lime changes the mineral composition of both perennial and annual pastures at anthesis in the high rainfall region of south-eastern Australia. However, those changes in pasture mineral composition do not always lead to improved mineral ratios and animal health indices. While the risk of some metabolic disorders in grazing livestock are reduced, but for others they increase. The primary drivers of improvements in important mineral ratios in favour of animal health and well-being were the increase in Ca and Na concentrations, serving to increased Ca:P ratio and decreased tetany index in this study. Kuusela^27^ suggested that a Ca:P ratio between 1.1 and 2.1 is ‘ideal’ and could improve the bone calcification. However, Ternouth^28^ found that livestock could tolerate dietary Ca:P ratios of more than 10:1 without ill effect provided that the P intakes meet animals requirements^29^. In the present study, the Ca:P ratio was significantly higher on the limed treatment than that on the unlimed treatment, but generally below 4:1. The higher Ca concentration with liming may reduce susceptibility to hypocalcaemia^17^, but higher Ca:P ratio might exert a decrease in bone formation and animal growth in a P deficient diet^30^.

The DCAD was high in both limed and unlimed pastures and increased with liming, which was not in line with the hypothesis. The pasture DCAD level in most years was > 20, likely due to high K and Na concentrations relative to S and Cl. Livestock grazing on high DCAD pasture/diets are prone to hypocalcaemia under conditions of high demand because of the inability of the body to mobilise Ca from bone by changing pH^29^. Research experiments have shown that the low DCAD in the diet can affect the endogenous synthesis or catabolism of 1,25-hydroxyvitamin D3 via activation of 1-α-hydroxylase^31^. It is expected that animals grazing on this pasture have lower concentration of 1,25-dihydroxyvitamin D3 due to high DCAD level, which may be an issue for locations poleward of the 34° latitudes with reduced solar radiation.

The extent to which the negative effects of a higher DCAD may be offset by a higher Ca content due to lime is an area warranting further investigation. The study of Masters et al*.*^29^ showed that offering low-DCAD supplement to reproducing ewes was less effective at improving Ca status when compared to an industry standard supplement. The authors suggested that the lack of an additional response could be due to the inefficiency of supplements in reducing the DCAD of total feed, brought about by limits to supplement intake. Thomas and Hargrove^32^ suggested that soil properties such as pH are likely to have an impact on herbage DCAD through effects on the availability of nutrients as low pH soils often have high soil Al, which reduces root growth and thus may affect the mineral nutrition of plants^33^. Concentrations of Al would be expected to be lower on limed compared to unlimed pastures, as in general, plants surviving in acidic soils contend with both Al and Mn toxicity and P deficiency^34^. In the present study, plant Al was less than 300 mg/kg on both limed and unlimed pastures, which is well below the maximum threshold assumed (1000 ppm) for livestock diets^35^.

There was a tendency that liming reduced plant Mg, P, S and Fe in the first liming cycle, but increased those minerals during the second liming cycle. The reason for those changes in availability over time is not clear. Our data showed that herbage concentrations of macro-minerals were highly correlated with exchangeable cations, with exchangeable Mg progressively increasing over the experimental period (M K Conyers unpublished data). The chance of potential hypomagnesaemia in livestock grazing on either perennial and annual pastures at this site was not high because of adequate levels of Mg (> 0.9 g/kg), Na (> 0.9 g/kg) and K (5–30 g/kg) together with a low tetany index and low K:(Na + Mg) index in the pasture, based on requirements of sheep and cattle^24^.

Many factors can impact both the supply and uptake of minerals, such as the availability of mineral nutrients in the rhizosphere, improvement in root growth allowing more soil to be explored^1^, altered uptake of nutrients per unit length of root^36^, and the moisture status of the soil^37^ for some minerals, such as P that moves by diffusion or S that moves via mass flow. The growth stage of the pasture also alters the relative content of minerals due to growth induced dilutions^21^. The action of lime in altering concentrations of macro elements in shoots could occur through change to the supply such as large quantities of Ca in lime competing with other base cations and changes in mineral availability in soils due to change in soil pH after liming^1,38^. This is in addition to dilution effects due to extra plant growth^20,36^. Consideration of all results indicates long-term liming would have beneficial role in reducing the incidence of hypocalcaemia and hypomagnesemia by improving Ca, Mg and Na concentration. These conclusions need to be validated with appropriate feeding or grazing studies.

Results showed that liming decreased the concentrations of some micro-nutrients including Cu, Zn and Mn. This is in broad agreement with the earlier evaluation conducted by Hayes et al*.*^22^, except that in that study the reduction in Cu concentrations was not observed in two (chicory and subterranean clover) of the six species tested. Manganese is available to plants in the reduced form, and Mn^2+^ can be toxic in some acidic soils. The application of lime on such soils lowers net Mn reduction, leading to lower Mn concentrations in shoots of pasture species^39^. Decreases of Cu, Mn and Zn concentration are notable because of their role on immunity and health by reducing oxidative stress and boosting innate and acquired immunity in dams and their offspring^40^. These minor elements play important roles in the superoxide dismutase pathway as the first line of the antioxidant defence, converting superoxide anion to hydrogen peroxide^41^. There may be implications for the plant in its ability to manage oxidative stress, as well as for the animal consuming it, when exposed to certain environmental stressors, such as heat stress, lamb marking, gestation, lactation stress and other stress when animal welfare is compromised. Copper deficiency is the most common trace element deficiency affecting beef cattle in southern Australia, as they have a higher dietary requirement for Cu than sheep^42^. Copper deficiency can be induced by low levels of Cu in the soil, high dietary intakes of molybdenum (Mo), S, Zn, Fe, cadmium (Cd) and Ca^42^. In the present study, the Cu concentrations on the limed treatment were lower than that on the unlimed treatment. Liming would seem to pose an elevated risk of Cu deficiency due to the lower concentrations of Cu in herbage, higher concentrations of Ca and Mo that may decrease absorption, and induced precipitation of Cu-hydroxides and/or Cu-carbonates^43^. There is an interaction between Cu, Mo, S and Fe, indicating that Cu absorption is dependent on these elements in the diet as well as Cu concentration^44,45^.

Lime would increase availability of Mo^1,46^, hence, concentrations of Mo would be expected to be higher on limed compared to unlimed pastures^47^, although they were not quantified in the present study. Copper concentrations in the range of 5–30 and Cu:Mo ratios > 2–4 are suggested to avoid Cu deficiency in livestock^47^. Further research on Cu nutrition and its interactions is recommended as the nutrient concentration of Cu in the present study did not meet the requirements for cattle or goats and failed to meet the requirements for late pregnancy or early lactating ewes. Molybdenum has been shown to have antagonistic and inhibitory effects on the Cu absorption in the rumen. The diet rich in Mo and S forms thiomolybdates which prevents Cu absorption and incorporation into plasma proteins^48^.

Deficiency of Zn can interrupt many biochemical and enzymatic processes in cattle, such as protein synthesis and carbohydrate metabolism, with risk of deficiency increasing in diets when Zn is below 40 mg/kg^49^. Most of the Zn concentration values observed in the present study were below that nominal risk threshold and its nutrient concentration did not meet the requirements for sheep at any stage considered in the present study, and often did not meet the requirements for cattle, or late pregnancy/early lactation goats. There is inconclusive evidence that other minerals, such as Ca, impede the absorption of Zn in cattle but high dietary Cd has been implicated. Cadmium was not measured in the present study, but it has been shown by Hayes et al*.*^22^ that Cd concentrations in subterranean clover decreased with lime. Therefore, Zn deficiency impacting livestock reproduction and health that is associated with lime application is more likely to be a direct result of reduced Zn availability in the herbage, rather than associated minerals impeding absorption^49,50^.

There were large year-to-year variations in herbage mineral concentrations, largely following rainfall distribution patterns. In general, pasture mineral concentrations tended to be higher under drought years (e.g. 1994, 2001–2003), but lower in the years with above-average rainfall (e.g. 1992, 1999 and 2000). Undoubtedly, this is due to a dilution of mineral concentrations in higher rainfall years when pasture growth is greater. Nevertheless, the study does demonstrate the large variability in mineral concentrations that can occur in a field environment highlighting the fact that lime alone cannot mitigate all risks of animal health disorders in all years, in the same way that risks of mineral deficiencies due to lime will not be constant from year to year.

In conclusion, long-term liming changed the pasture mineral profile on both perennial and annual pastures. Liming increased the Ca:P ratio and decreased the tetany index, potentially improving animal health outcomes. However, the likelihood of hypocalcaemia may remain as an issue with high DCAD levels in herbage, increasing the risk of vitamin D deficiency and bone disorders in livestock. Reduced concentrations of some micro elements, such as Mn, may also potentially impair antioxidant capacity in livestock.

## Methods

### Site description and experimental design

The experiment was conducted for 12 years from 1992 to 2003 on the property ‘Brooklyn’, at Book Book (147° 30′ E, 35° 23′ S; 40 km south-east of Wagga Wagga) where long-term average annual rainfall was 650 mm. The soil was a subnatric Yellow Sodosol with some red phases across the site^51^. The average pH in 0.01 M CaCl_2_ (pH_Ca_) was 4.0 and 4.2 in the 0–10 and 10–20 cm soil depths, respectively, with aluminium (Al) comprising 31% and 43% of total exchangeable cations in the corresponding depths.

A long-term experiment, known as MASTER (Managing Acid Soil Through Efficient Rotations) was established in 1992^8^. Four treatments were chosen for this study, namely, perennial pastures with or without lime (PP+ and PP−) and annual pastures with or without lime (AP+ and AP−). The experiment was then embedded with 6 liming phases for PP+ and AP+, with 2 plots per phase, effectively 12 replicates for the limed treatments. For PP− and AP−, there were only 6 replicates for each treatment. There were 36 plots in total with plot size as 30 m × 45 m with 10 m laneways. The perennial pasture was sown to phalaris (*Phalaris aquatica* L.), cocksfoot (*Dactylis glomerata* L.), lucerne (*Medicago sativa* L.) and subterranean clover and the annual pasture was sown to annual ryegrass (*Lolium rigidum* Gaudin.) and subterranean clover (Table 3). Lucerne and subterranean clover were inoculated with recommended rhizobia. Phosphorus (P) was applied at 30 kg P/ha at sowing in 1992, and surface-spread at 15 kg P/ha in autumn every year over the life of the experiment. The pastures were rotationally grazed as per protocols described in Li et al*.*^9^ and Li et al*.*^12^.

**Table 3:** Treatment description and pasture species mixes at establishment in 1992.

Treatment^a^	Species	Variety	Sowing rate
Perennial pastures (PP+ vs PP−)	Phalaris (*Phalaris aquatica* L.)	Australian	0.5 kg/ha
		Holdfast	1.0 kg/ha
	Cocksfoot (*Dactylis glomerata* L.)	Currie	1.0 kg/ha
	Lucerne (*Medicago sativa* L.)	Aurora	3.0 kg/ha
	Subterranean clover (*Trifolium subterraneum* L.)	Junee	1.5 kg/ha
		Goulburn	1.5 kg/ha
		Trikkala	1.5 kg/ha
Annual pastures (AP+ vs AP−)	Annual ryegrass (*Lolium rigidum* Gaudin.)	Wimmera	2.0 kg/ha
	Subterranean clover (*Trifolium subterraneum* L.)	Junee	2.5 kg/ha
		Goulburn	2.5 kg/ha
		Trikkala	2.5 kg/ha

^a^PP+ versus PP−, Perennial pastures with or without lime application; AP+ versus AP−, Annual pastures with or without lime application.

Superfine lime (70% ≤ 250 μm, neutralising value 98%, Omya Southern Pty Ltd) was used in the experiment. Lime was applied on a 6-year cycle with the first year when lime was applied as phase 1 and the last year before re-liming as phase 6. However, all limed treatments were limed in April 1992 with different lime rates (3.4–4.1 t/ha) depending on the designated phases. The initial lime application was incorporated into the 0–10 cm soil layer and the maintenance lime was top-dressed at the start of phase 1 for a given plot. The target was to maintain the average pH at 5.5 in the 0–10 cm depth. Further details of the liming regime were reported in Li et al*.*^2^.

### Plant sampling and analysis

Pasture pluck-samples were taken at anthesis each year to mimic sheep grazing by avoiding the less digestible and unpalatable stem fraction and reflecting the composition of the sward at the time of sampling. The pasture samples (~ 500 g from each plot at 20 locations) were kept cool in an insulated box in the field for transport to the laboratory where they were dried at 70 °C for 48 h in a dehydrating oven. The samples were initially ground to pass a 2-mm sieve in a plant grinder, and then ground into a fine powder in a puck mill. Subsamples were pressed into 32-mm pellets using a hydraulic press. The mineral content of the pasture was evaluated for macro minerals Ca, Mg, K, Na, P, S, Cl and Si; and micro minerals Al, Mn, Cu, Fe and Zn with a Philips 1404 X-ray fluorescence spectrometer (Philips, Almeto, the Netherlands) using a dual anode Sc-Mo tube^52^.

Pasture mineral ratios including Ca:P; K:Na and K:(Na + Mg) ratio, and indices, including tetany index [K:(Ca + Mg)] and the dietary cation anion difference [DCAD = (Na + K) − (Cl + S)] in milli-equivalent were calculated^18^.

### Statistical analysis

Pasture mineral concentrations for macro elements (Ca, K, Mg, Na, P, S, Cl and Si), micro elements (Al, Mn, Cu, Fe and Zn), mineral ratios [Ca:P, K:Na and K:(Na + Mg)] and indices (tetany index and DCAD) were spline-fitted using ASReml-R^53^. The fixed factors were lime, pasture type, sampling year and associated two-way and three-ways interactions. Random factors were replicate, the spline component of sampling year, and associated interactions. All random terms were included in the initial model, but terms that failed to achieve statistical significance at *P* = 0.05 were excluded from the final model. The fixed effects were tested using the Wald statistics, and the random effects were tested using the residual maximum likelihood ratio test. Correlations between pasture macro elements were computed using ‘GGally’ package in R environment^54^.

### Statement of research involving plants

The authors wish to affirm our commitment to compliance with relevant institutional, national, and international guidelines and legislation governing the collection and utilisation of plant materials.

## Data Availability

The data that support the findings of this study are available from the corresponding author upon reasonable request.
